# Freshwater Sponges Have Functional, Sealing Epithelia with High Transepithelial Resistance and Negative Transepithelial Potential

**DOI:** 10.1371/journal.pone.0015040

**Published:** 2010-11-29

**Authors:** Emily D. M. Adams, Greg G. Goss, Sally P. Leys

**Affiliations:** Physiology, Cell and Developmental Biology Group, Department of Biological Sciences, University of Alberta, Edmonton, Alberta, Canada; University of Queensland, Australia

## Abstract

Epithelial tissue — the sealed and polarized layer of cells that regulates transport of ions and solutes between the environment and the internal milieu — is a defining characteristic of the Eumetazoa. Sponges, the most ancient metazoan phylum [1], [2], are generally believed to lack true epithelia [3], [4], [5], but their ability to occlude passage of ions has never been tested. Here we show that freshwater sponges (Demospongiae, Haplosclerida) have functional epithelia with high transepithelial electrical resistance (TER), a transepithelial potential (TEP), and low permeability to small-molecule diffusion. Curiously, the *Amphimedon queenslandica* sponge genome lacks the classical occluding genes [5] considered necessary to regulate sealing and control of ion transport. The fact that freshwater sponge epithelia can seal suggests that either occluding molecules have been lost in some sponge lineages, or demosponges use novel molecular complexes for epithelial occlusion; if the latter, it raises the possibility that mechanisms for occlusion used by sponges may exist in other metazoa. Importantly, our results imply that functional epithelia evolved either several times, or once, in the ancestor of the Metazoa.

## Introduction

A sealed epithelium is necessary to control the internal milieu of animals but it is particularly important in fresh water, where osmotic stress and loss of ions can disrupt cell physiology [6]. Epithelia are considered to have first evolved in ancestors of the Cnidaria, so sponges (Porifera), which diverged up to 200 million years prior to other metazoans [2], are the only multicellular animals thought to lack true epithelia, and it is widely considered that the environment can circulate through the sponge body [3], [4], [5], [7]. The most current phylogenomic analyses of animal relationships place sponges as most basal [1], [5], [8], but other hypotheses suggest ctenophores or placozoans are the earliest branching group [9], [10], both of which are considered to have true occluding epithelia [11], [12], [13], [14]. In these scenarios sponges must have lost the ability to form epithelia with occluding junctions.

Epithelia are composed of simple or stratified layers of polarized cells that are connected by intercellular junctions. In most animals desmosomes and adherens junctions (AJs) control whole tissue integrity [4], while semi-permeable occluding junctions such as tight junctions (TJs) or septate junctions (SJs) selectively control molecule and ion passage between cells [15]. Epithelia are also usually supported by an extracellular basement membrane (the basal lamina), which prevents dedifferentiation and migration of cells into the mesenchyme [16]. Although epithelial characteristics are often rigidly defined, epithelia are actually dynamic structures with variable integrity and tightness depending on physiological requirements throughout the body or during ontogeny [17]. Moreover the recent discovery of new types of cell junctions in vertebrate tissues [18] demonstrates that the characterization of cell-cell interactions is still a developing field: in some instances molecules are absent where the morphology shows a junction; in others, junctional molecules are present but no junction can be seen by electron microscopy [18]. Sealing and adhesion may therefore occur in the absence of classical occluding and adherens molecules or junctions.

In the early 20th century sponges were considered to be syncytial because the limits of cell boundaries were often very hard to discern by light microscopy [19], [20]. Later transmission and scanning electron microscopy confirmed that all sponges except Hexactinellida are cellular; glass sponges are unusual because their tissues fuse during early embryogenesis to form syncytia [21] which allow the propagation of electrical impulses through the whole animal [22]. All other sponges are cellular, and as far as it is known there are no aqueous junctions (e.g. gap junctions) between cells; therefore cellular sponges do not propagate electrical signals.

The surface of cellular sponges is formed by a single layer of thin, pentagonal cells called pinacocytes (pavement cells) that enclose a collagenous mesohyl (middle layer) containing mobile amoeboid cells. Sponge pinacoderms are polarized by the unilateral secretion of proteins and express the polarity genes *Frizzled*, *discs large, Par*, and *Crumbs* [5], [23], [24]. The membranes of adjacent pinacocytes are typically closely apposed, separated by a small 15 nm intercellular gap [11]. Desmosomes and junctions with septae have been found between cells in all sponges [17], and although cadherins and catenins are present in the sponge genome [5], only a few of the genes associated with conventional SJs appear to be present (e.g., *discs large* [5] and *neurexin* [25]).The most convincing images of junctions with dense septae are between cells that secrete spicules in the Calcarea [26], and this has given rise to the opinion that septate junctions only arise transiently to isolate regions for spiculogenesis [27], [28]. But regions of membrane fusion [29], [30] also occur in sponges, and some tight junction-related genes (e.g., members of the tetraspanin and MAGUK family of proteins) are in the *Amphimedon* genome [5], [24] and are expressed in the pinacoderm of other demosponges [31].

Among Porifera, the homoscleromorphs (Homoscleromorpha) are distinct in possessing a clear basement membrane with immunoreactivity to type IV collagen [32]. Although other sponges appear to lack an obvious basement membrane, a structurally similar network containing fibronectin and a homolog of type IV collagen underlies pinacoderms [17], [33], [34], and integrins are localized at the basal cell surface [5], [35]. Thus sponges as a group possess the components for a functional extracellular matrix capable of supporting an epithelium, but only homoscleromorphs possess a typical basement membrane, the importance of which is unclear.

We recently demonstrated that freshwater sponge pinacocytes are static over time [17] and that glutaminergic signalling controls a coordinated inflation and contraction behaviour – in effect, a sponge ‘sneeze’ [36]. Other demosponges as well as calcisponges (Calcarea) and homoscleromorphs are also able to contract periodically [37]. These behavioural traits are evidence that pinacocytes must establish stable attachments for communication and coordination. But the ultimate demonstration of an epithelium is the ability to regulate selectively the passage of solutes: that is, its ability to seal and create a transepithelial potential [4]. Although all animals must have some ability to regulate ion balance in the internal matrix, freshwater animals, including freshwater demosponges, must have experienced strong evolutionary pressures to regulate an internal environment, and therefore this group provides a particularly valuable model to study the emergence of key features of functional epithelia.

To determine whether freshwater sponge epithelia can seal and generate an electrochemical potential we took advantage of the ability of sponge cells to form aggregates from dissociated cells [38] and hatch from gemmules (cysts of undifferentiated cells), enabling us to grow cell cultures on permeable membranes. We show that *Spongilla* tissue has transepithelial resistance, is impermeable to the passage of small tracer molecules, and has a negative transepithelial voltage and therefore functions in all aspects as a true epithelium. This is the first demonstration of functional epithelia outside of the Eumetazoa sensu stricto.

## Results

**Figure 1:**
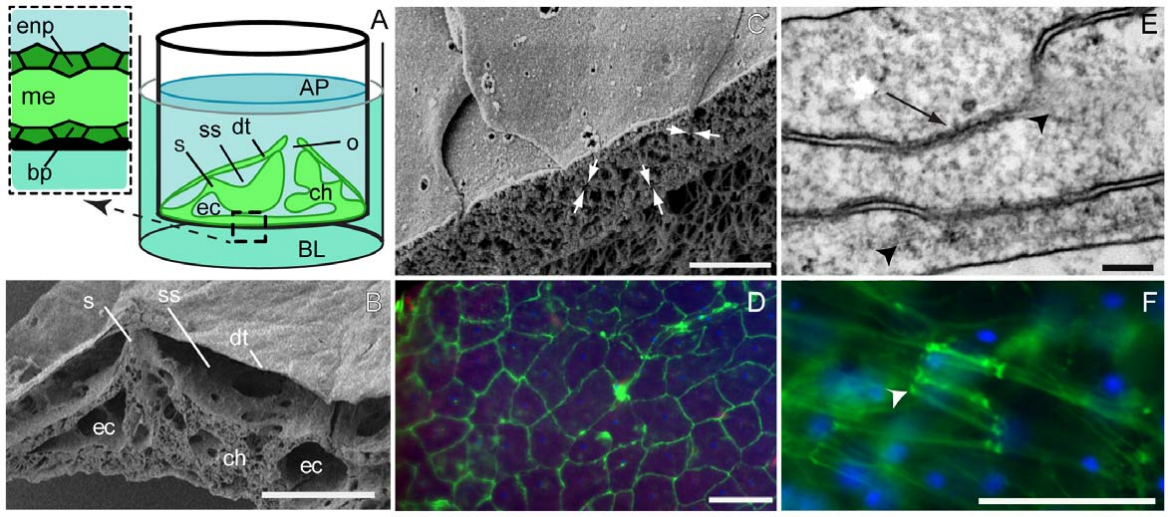
Structure of the sponge epithelium. (**A**) Diagram of the experimental chamber, and (**B**) scanning electron microscopy (SEM) of sponge tissue showing: the bilayered dermal tissues (dt), suspended over the subdermal space (ss) by shafts of spicules (s); the choanosome (ch) with the choanocyte pumps; excurrent canals (ec); and an osculum (o). For transepithelial recordings, apical (AP) and basolateral (BL) compartments were effectively separated by two confluent cell layers - the basopinacoderm (bp) and endopinacoderm (enp) - surrounding a thin mesohyl (me) (inset); scale bar b, 100 µm. (**C**) SEM of the dermal tissue shows exopinacocytes are very close to one-another (opposing arrows show cell-cell spacing); scale 1 µm. (**D**) Fluorescent labelling of the basopinacoderm with the steryl dye FM 1–43 highlights the borders of cells showing that a confluent layer of cells covered culture membranes; scale 10 µm. (**E**) Transmission electron microscopy of exopinacocytes shows tight membrane apposition between adjacent cells (arrow). These areas were associated with 10 nm diameter cytoskeletal fibres (arrowheads); scale 200 nm. (**F**) Fluorescent labelling using phallacidin reveals dense plaques of actin (arrowhead) between cells; nuclei, blue; scale, 5 µm.

*Spongilla lacustris* cells formed confluent layers 3–5 days after seeding culture membranes with either aggregates or gemmules. We measured the transepithelial electrical resistance on either side of the sponge tissue using chopstick electrodes inserted into the upper (apical) and lower (basolateral) chambers (Figure 1A). The electrode in the apical chamber was set at zero while the electrode in the basal chamber recorded the resistance/voltage. Scanning electron microscopy showed that the cultures had all the attributes of a typical sponge (Figure 1B) with closely apposed exopinacocytes in the dermal tissue or surface of the sponge (Figure 1C), canals lined by endopinacocytes, choanocyte chambers and an osculum or chimney-like vent; they were therefore good models of native sponge tissues. The sponge basopinacoderm (which contacts the culture membrane directly) is formed by exopinacocytes which secrete proteins that form an attachment to surfaces. Since the canal system is contiguous with the external environment, resistance to ion passage occurred through two simple cell layers, the endopinacoderm and basopinacoderm (Figure 1A inset). The basopinacocytes completely covered the membrane, forming a mosaic of cells that resembled vertebrate epithelial cell cultures (Figure 1D). Punctate regions between exopinacocytes were associated with 10 nm diameter actin fibres that extended into the cytoplasm (Figure 1E). These regions corresponded to actin-dense plaques found in the exopinacocyte layers of both *S. lacustris* and *Ephydatia muelleri* (Figure 1F).

**Figure 2:**
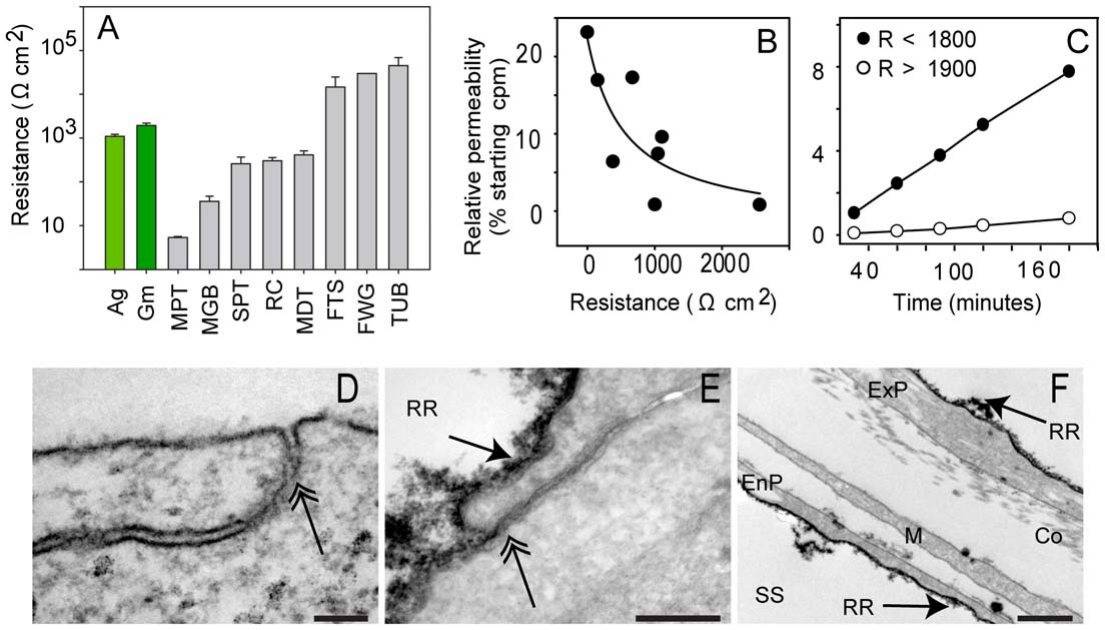
Electrical resistance and impermeability of *S. lacustris* epithelia. (**A**) The resistance to ion passage of *Spongilla* epithelia derived from aggregates (Ag) (1098.5 Ω cm^2^±671.5 s.e. n = 33) and gemmules (Gm) (1932.5 Ω cm^2^±253.5 s.e. n = 22) was comparable to the resistance of epithelia from vertebrate tissues (MPT =  mammal proximal tubule, MGB =  mammal gall bladder, SPT =  salamander proximal tubule, RC =  rabbit colon, MDT  =  mammal kidney distal tubule FTS =  frog/toad skin; FWG =  freshwater fish gill; TUB =  toad urinary bladder). (**B**) Permeability to ^3^H inulin decreased with increasing resistance of sponge cultures. (**C**) ^3^H inulin gradually accumulated on the basolateral side of cultures with low resistance (solid circles, <500 Ω cm^2^), but was excluded by high resistance epithelia (open circles, >800 Ω cm^2^). (**D**) Regions of membrane fusion, as seen by transmission electron microscopy (TEM) were common near the most apical point of cell contact in exopinacocytes; scale 100 nm. (**E**) Ruthenium red (RR, the dark precipitate) was excluded from paracellular clefts (double arrow); scale, 200 nm. (**F**) RR was excluded by exopinacocytes (ExP) and endopinacocytes (EnP) from the collagenous (Co) internal mesohyl containing mesohyl cells (M). The tracer entered the aquiferous system though ostia and therefore coated the endopinacoderm (En) roof of the subdermal space (SS); scale, 500 nm.

Cultures that were confluent across the membranes had resistances (R) above 300 Ω cm^2^ and up to 5500 Ω cm^2^, comparable to the resistance of vertebrate epithelia (Figure 2A). Cultures formed from gemmules had a higher resistance (mean 1932.5 Ω cm^2^±253.5 s.e., n = 22) than those formed from aggregates (mean 1098.5 Ω cm^2^±671.5 s.e., n = 33) (Figure S1), possibly because the cultures made from gemmules contained more cells. Initial resistances of the sponge epithelium were even higher, but dropped when we began recording because it was necessary to add salt (<0.1% w/v NaCl) to the medium so that the instrument could measure a current. We found that the resistance stabilized at 30% of the initial value after 10 minutes; here we report the resistance after two minutes in the recording medium.

Epithelial resistance varies by orders of magnitude between different tissue types and animals. Freshwater fish gill epithelia have one of the highest recorded resistances at up to 40,000 Ω cm^2^ (Table S1). However tissues considered to be true epithelia, including proximal kidney tubule, can also have very low resistances when they are associated with nutrient or waste exchange [39]. Leakiness has been loosely correlated with the depth of the junctional complex in the epithelium [40]. In the mouse proximal kidney tubule, a model leaky epithelium, cells appear “suspended in the urinary space” and have convoluted interdigitations of their membranes that make only small connections with each other and the capillary wall [41]. In contrast, model tight epithelia such frog skin and fish gill have junctional complexes that form extensive connections around the circumference of the cell. At the molecular level in vertebrates the presence of claudin 2 has recently been shown to correlate directly with leakiness and ability to re-absorb ions [42]. Nevertheless, in the absence of claudins in invertebrates, tightness and leakiness cannot be defined at molecular level. Freshwater sponges represent a mid-range of resistance: typically a resistance of 500–1500 Ω cm^2^ is associated with a tight epithelium [39].

Sponge cultures with a high electrical resistance excluded the flux of a 5 kD 1.5 nm diameter ^3^H-inulin tracer (Figure 2B). For cultures with the highest resistance, only 0.8% of the tracer was able to pass through after three hours (Figure 2C). A higher molecular weight tracer (^14^C PEG MW = 7000) was also excluded from passage through sponge cultures that had a high resistance (Figure S2). A potential site for this occlusion is in regions where the membranes of adjacent cells appear fused (Figure 2D). The electron dense tracer ruthenium red (MW = 859) was excluded from the paracellular space (Figure 2E). The tracer was not able to enter the sponge mesohyl (Figure 2F).

**Figure 3:**
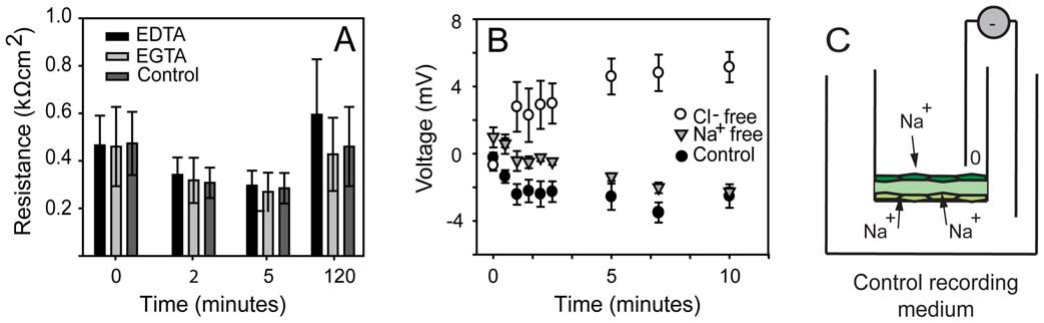
Ionic basis of occlusion in sponge epithelia. (**A**) Effect of EGTA or EDTA treatment on resistance. All cultures lost resistance in the recording medium and recovered high resistance after 2 hr in the culture-medium. (**B**) Voltage across the epithelium was negative in the normal culture medium and in sodium-free medium and positive in chloride-free medium. (**C**) Schematic showing the uptake of sodium in normal recording medium to generate a negative potential.

To better understand the characteristics of ion occlusion by sponge epithelia we treated cultures with the divalent cation chelators EGTA and EDTA. In the absence of calcium, we expected that, as in vertebrate models, disruption of calcium-dependant adhesion would lower resistance. But neither chelator reduced the resistance of sponge epithelia more than control cultures in recording medium alone (Figure 3A). Both controls and EGTA-treated cultures recovered high resistance after 2 hours in culture medium. These results could suggest that the mechanism of occlusion in freshwater sponges is not calcium dependent, but because ruthenium red was occluded from even the outermost region of the cell-cell junctions it is more likely that in *Spongilla* occluding junctions occur apical to the adherens junctions, so that chelators would not affect the integrity of the occluding junction. A similar situation is seen in *Drosophila*, where the subapical region (SAR, apical to the zonula occludens) has protein complexes that co-localize with tight junctions from vertebrate cells [43]. Alternatively, in sponge epithelia the protein complexes involved in adhering and occluding junctions may not interact as they do in vertebrate models.

The ability to generate an asymmetrical electrochemical potential is a property of all epithelia and confirms that sealing junctions must be present [4]. In symmetrical recording medium (the same solution in both compartments) the sustained transepithelial electrical potential across *Spongilla* cultures was slightly negative (∼ −3 mV, Figure 3B), indicating that active transport occurs between the sponge and the water. To determine the source of the net negative potential we used symmetrical chloride-free or sodium-free media. In Cl-free media the voltage was positive (Figure 3B), indicating a net loss of chloride into the apical compartment. In the absence of sodium voltage was slightly less negative than in the control recording medium (Figure 3B). Although the voltage may arise from net loss of ions it is unlikely that in normal freshwater sodium or chloride would be lost from tissues. Therefore the negative potential in the normal recording medium is most readily explained by active net uptake of sodium (Figure 3C). These results indicate that under normal ionic conditions the apical sponge tissue (endopinacoderm and dermal tissues) is tight, as supported by the lack of tracer flux. Most importantly, the results show that sponge epithelia, like epithelia of other animals, maintain an electric potential.

## Discussion

In this study we show that epithelia in *Spongilla lacustris* have relatively high electrical resistances, can impede the passage of small molecules, and control their membrane potential by transport of ions. Our results provide the first demonstration of high resistance and occlusion of small tracers, and suggest that ion transport also occurs in freshwater sponge tissues; these three features are key indices of epithelial function in vertebrate and invertebrate *in vivo* and *in vitro* systems. Occlusion is likely localized at regions where adjacent cell membranes are in tight contact (Figure 2D). Alternatively, proto-occluding junctions could form a diffuse network along the intercellular cleft, as occurs in the epithelia of developing vertebrates [18]. If so, the resistance of sponge epithelia to ion transport and ability to occlude small molecules may be related to the “membrane spacing factor” defined by Green and Bergquist [11] and would also control the regular cell spacing between pinacocytes in the absence of septae or other electron dense proteins.

The physiology and morphology described here indicates that sponge epithelia are functionally similar to animal epithelia with tight junctions. The tracer studies show exclusion of 5 kDa molecules; the morphology shows regions of tight membrane apposition and exclusion of ruthenium red, a very small tracer (0.85 kDa) that is commonly used to indicate the location of tight/septate junctions in tissues. Our focus here was on the physiology of sponge epithelia, and we are now examining EST libraries for the presence of Neurexin IV family genes or orthologs of occludins or claudins in other sponges. But given that these genes appear to be absent from the *Amphimedon* genome [5], either *Amphimedon* has secondarily lost genes for occlusion, or the mechanism of occlusion used by sponges has evolved independently from that used by other metazoans. If so, then unknown molecular components may have been responsible for ionic regulation in evolutionarily ancient organisms (e.g. the last common ancestor of animals) and may have been retained in their descendants. Further study of basal node metazoans and comparisons with known vertebrate systems may reveal overlooked or hidden components of epithelial ion regulation and pathophysiologies in ‘higher’ animals.

Not all sponges are as easy to culture, but it would be informative to try to culture homoscleromorph sponges to measure the resistance and exclusion of ruthenium red and other molecules by their epithelium. This may reveal physiological differences due to the presence of type IV collagen in the only known sponge group with a true basement membrane. Our attempts to culture tissue from the marine haplosclerid *Haliclona* cf *permollis* were impeded by the presence of ciliates which eventually destroyed the cells, but cultures should be possible either from marine sponges that produce gemmules, or from other species less prone to infestation by ciliates. Nevertheless, the similarity in morphology of other demosponge epithelia in general [17]suggests that the epithelia of marine demosponges may also seal. Freshwater sponges have arisen from marine haplosclerid sponges [44], which are common in shallow and intertidal habitats worldwide, environments that experience daily changes in salinity and humidity. Therefore marine haplosclerids may also be expected to regulate the passage of solutes. One measurement from our initial work suggested that *Haliclona* tissue also generated a resistance that, as expected, was somewhat lower than that recorded from the freshwater sponge.

**Figure 4:**
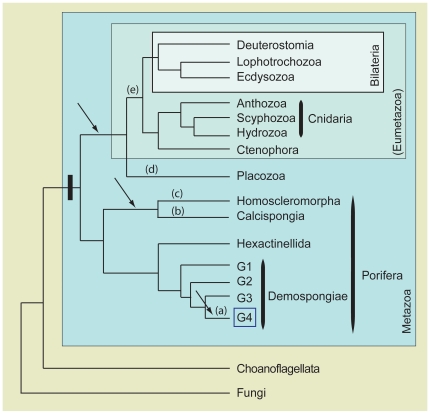
Sponge phylogeny and the evolution of epithelial characters. A phylogeny of Metazoa adapted from Philippe et al 2009 which considers Porifera monophyletic. Sponge phylogeny is adapted from [48]. Freshwater sponges belong to the group indicated with a box (G4). Letters indicate evidence of epithelial characters: (a) transepithelial resistance (TER) and transepithelial potential (TEP) in freshwater sponges; (b) septate junctions in calcareous sponges; (c) a basement membrane in homoscleromorphs; (d) absence (loss?) of a basement membrane in placozoans; (e) true epithelia with septate junctions, a basal lamina, TER and TEP in cnidarians and bilaterians. Arrows indicate three potential origins of epithelia; the solid bar indicates the most parsimonious scenario for the origin of epithelia.

The fact that sponges have polarized epithelia that seal and control the passage of solutes implies that at least freshwater demosponges, and likely other sponges, have functional tissues. These results provide the first concrete evidence to refute the concept that sponges tissues are only transiently sealed [27], [28], or “have no internal milieu because the environment can circulate through the body” [4]. This new perspective has relevance to our understanding of the Eumetazoa, which was originally defined to encompass organisms that have a mouth and gut and tissue level organization [45]. If sponges have functional epithelia, and epithelia are usually considered to be tissues, then the presence of tissues can no longer be used as a eumetazoan character. But more importantly, if epithelia exist in sponges, then either demosponges have evolved epithelia independently, or epithelia arose with the evolution of the first multicellular animals (Figure 4). We suggest that the first metazoans must have had a way of controlling their internal milieu in order for signal transduction pathways to function, and that a sealed epithelium was therefore one of the first defining features of multicellular animals.

## Materials and Methods

### Collection, culturing and fixation of tissues

Tissue and gemmules of the freshwater demosponges *Spongilla lacustris* (Linnaeus 1759) and *Ephydatia muelleri* (Lieberkuhn 1955) were collected from Sarita and Rosseau Lakes, Vancouver Island, B.C., Canada. We disassociated 10 cm^2^ pieces of *S. lacustris* through 100 µm Nitex mesh into artificial freshwater (M-medium) [46]. Aggregates (∼5 mm^3^) formed after a week of gentle rotation at 15°C and were seeded into Millicell hanging cell-culture inserts (polyethylene terephthalate, 0.4 µm pores, 0.33 cm^2^ filter area) (Millipore™, Billerica, MA, USA). Alternatively, we seeded 25 gemmules into each insert. Inserts were suspended in a two litre basin of M-medium with aeration to generate water circulation. Fixation for SEM and epifluorescence were described previously [47].

### TER

We used an epithelial volt-ohmmeter (EVOM; W.P.I., Sarasota, FL) to measure the transepithelial electrical resistance across culture inserts in 24-well Falcon culture dishes with 0.25 ml M-medium in the apical and 1.5 ml M-medium in the basal sides (Table S2). Because M-medium had insufficient ions to record current we added 0.06–0.099% w/v NaCl to the M-medium. All recordings were corrected for the empty Millicell insert in each medium. For Ca^2+^ chelation, we added 0.5 mM EGTA and 1 mM NMDG-Cl to the recording medium without CaCl_2_. For Ca^2+^ and Mg^2+^ chelation, we added 0.5 mM EDTA, 2 mM NMDG-Cl and 1.5 mM NMDG-SO_4_ to recording medium without MgSO_4_, CaCl_2_, and MgCl_2_. Chemicals were obtained from Sigma (Oakville, ON) unless otherwise noted.

### Paracellular molecule flux

We added 1 µCi ml^−1^ of [^3^H] inulin or [^14^C] PEG to the apical side of *S. lacustris* cultures. At t = 0 10 µl was sampled from the apical side to determine initial value; thereafter 100 µl was sampled from the basolateral side every 30, 60, 90, 120 and 180 min. Samples were stored at 4°C, mixed with 4 ml of ACS Fluor scintillation counting fluid (Fisher, Ottawa, ON) and radioactivity was measured with a Beckman LS 6000 scintillation counter. For electron microscopy of tracer flux, tissues were fixed for 1 hour in 2% glutaraldehyde with 1 mg ml^−1^ ruthenium red (RR) in 0.02 M cacodylate buffer, rinsed three times in buffer and post-fixed in 1% OsO4 with 1 mg ml^−1^ RR in buffer. Following dehydration in ethanol, the tissue was embedded in Spurs (Electron Microscopy Services, Hatfield, PA, USA); thin sections (60–70 nm) were cut on a Leica Ultracut T ultramicrotome, stained with uranyl acetate and lead citrate and viewed in a Morgagni FEI TEM.

## Supporting Information

Figure S1 **Box plots showing the resistance of tissue cultures from** ***Spongilla lacustris*** **gemmules (mean 1932.5 Ω cm^2^, n = 22, s.e. 253.5) and aggregates (mean 1098.5 Ω cm^2^, n = 33, s.e. 116.9).** Mean =  dashed line, Median  =  solid line, all outliers are shown.
(TIF)

Figure S2 **C^14^-PEG permeability through cultures from aggregates; legend shows the resistances of the different preparations in Ω cm^2^.** Preparations with a higher resistance (977 Ω cm^2^, 908 Ω cm^2^) excluded more radioactive tracer than cultures with a low resistance (231 Ω cm^2^). In all cases the amount of tracer that could pass through the sponge tissue did not increase over time.
(TIF)

Table S1 **Data sources for meta-analysis of resistance of epithelial cultures.** (Adapted from Boulpaep & Seely 1971, Claude & Goodenough 1973, and Powell 1981).
(DOC)

Table S2 **Salt concentrations in culture media.**
(DOC)
