# Supplementary figures and images for: Freshwater Sponges Have Functional, Sealing Epithelia with High Transepithelial Resistance and Negative Transepithelial Potential
Source: PLoS One. 2010 Nov 29;5(11):e15040. doi: 10.1371/journal.pone.0015040 (PMC2993944; doi:10.1371/journal.pone.0015040)

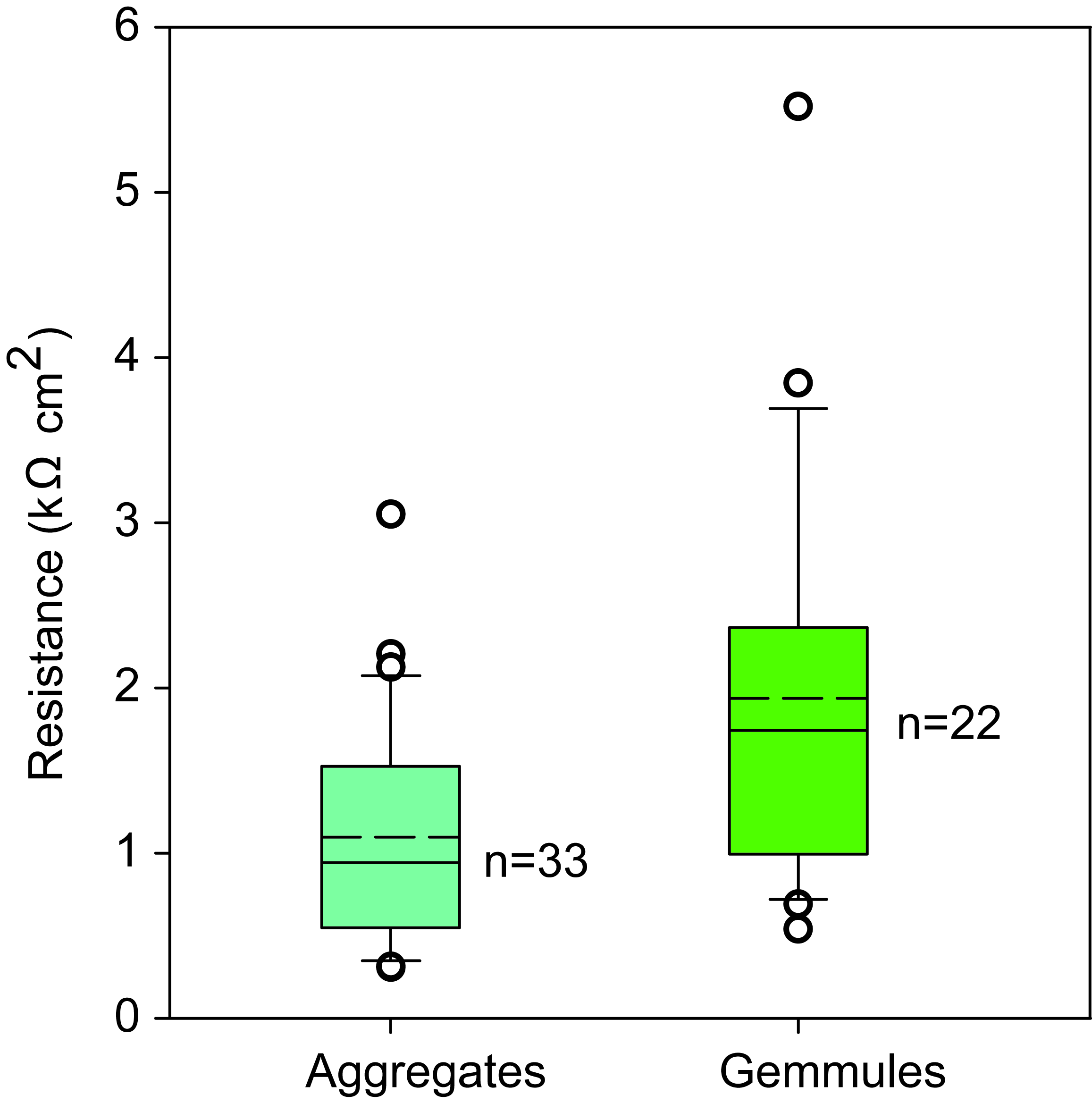

Supplement: Figure S1 — Box plots showing the resistance of tissue cultures from Spongilla lacustris gemmules (mean 1932.5 Ω cm2, n = 22, s.e. 253.5) and aggregates (mean 1098.5 Ω cm2, n = 33, s.e. 116.9). Mean = dashed line, Median = solid line, all outliers are shown. (TIF) [file pone.0015040.s001.tif]

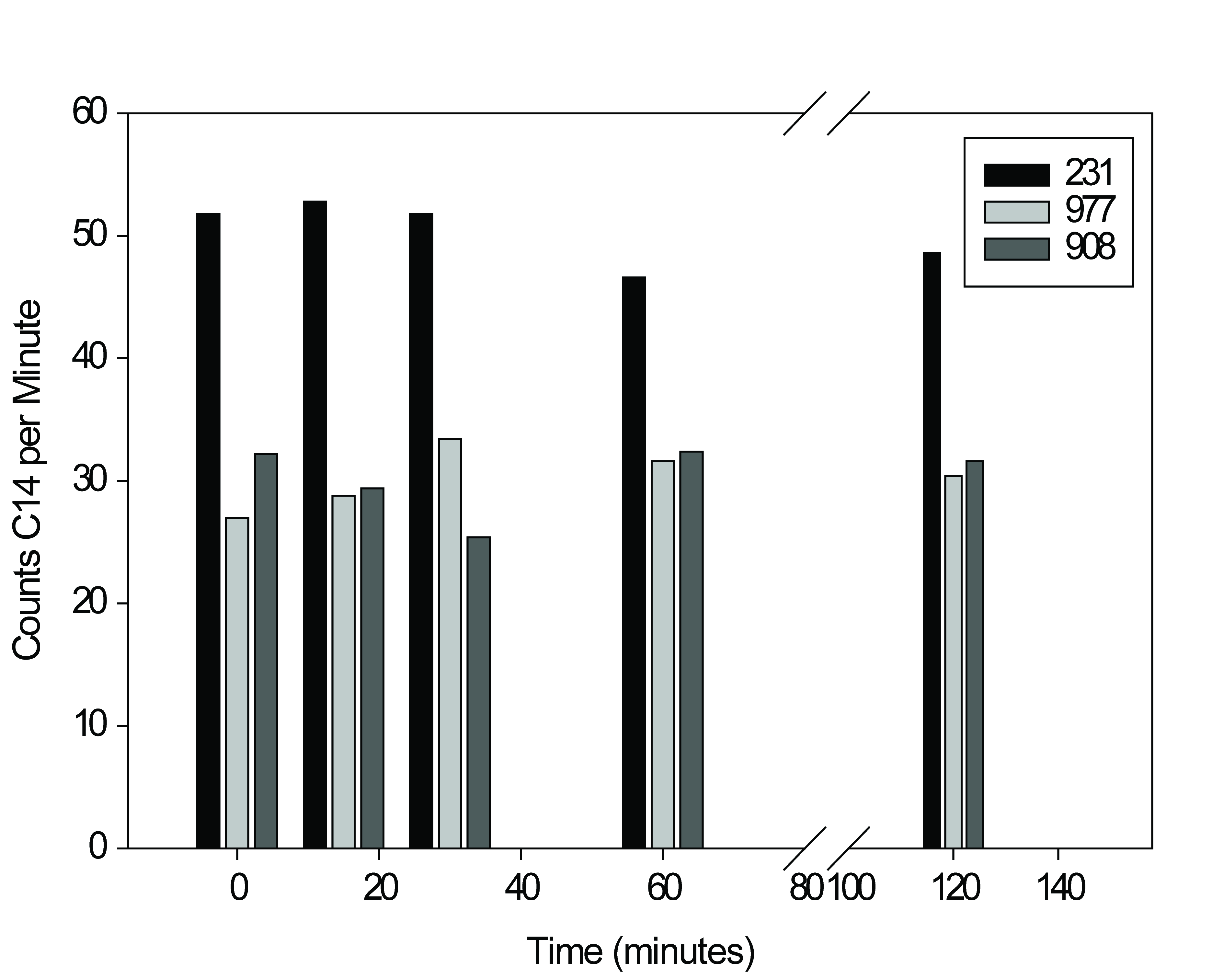

Supplement: Figure S2 — C14-PEG permeability through cultures from aggregates; legend shows the resistances of the different preparations in Ω cm2. Preparations with a higher resistance (977 Ω cm2, 908 Ω cm2) excluded more radioactive tracer than cultures with a low resistance (231 Ω cm2). In all cases the amount of tracer that could pass through the sponge tissue did not increase over time. (TIF) [file pone.0015040.s002.tif]
