# Supplementary material for: Freshwater Sponges Have Functional, Sealing Epithelia with High Transepithelial Resistance and Negative Transepithelial Potential
Source: PLoS One. 2010 Nov 29;5(11):e15040. doi: 10.1371/journal.pone.0015040 (PMC2993944; doi:10.1371/journal.pone.0015040)
Supplement: Table S1 — Data sources for meta-analysis of resistance of epithelial cultures. (Adapted from Boulpaep & Seely 1971, Claude & Goodenough 1973, and Powell 1981). (DOC) [file pone.0015040.s003.doc]

| **Tissue Type** | **Resistance** | **Reference** |
| --- | --- | --- |
| **Dog Proximal Tubule** | 6.96 | Lutz MD *et al.* (1973) *Am. J. Physiol.* **225,** 729-73 |
| **Dog Proximal Tubule** | 5.6 | Boulpaep EL & Seely JF (1971) *Am. J. Physiol.* **221,** 1084-1096 |
| **Rat Proximal Tubule** | 4.9-5.7 | Hegel U, Frömter E & Wick T (1967) *Arch. Ges. Physiol.* **294,** 274-290 as cited in: Boulpaep EL & Seely JF (1971) Ibid. |
| **Necturus Proximal Tubule** | 260 | Guggino WB (1982) *J. Membr. Biol.* **67,** 143-154 |
| **Necturus Proximal Tubule** | 70 | Boulpaep EL (1969) as cited in: Boulpaep EL & Seely JF (1971) Ibid. |
| **Necturus Proximal Tubule** | 450 | Anagnostopoulos T *et al.* (1980) *J. Gen. Physiol.* **75,** 553-587 |
| **Human Gallbladder** | 58 | Rose *et al.* (1973) *Am. J. Physiol.* **224,** 1320-1326 |
| **Rabbit Gallbladder** | 20 | Henin et al. (1977) *J. Membr. Biol.* **34**, 73-91 |
| **Rabbit Gall bladder** | 30 | Frömter E & Diamond J (1972) *Nat. New Biol.* **235,** 9. |
| **Frog skin** | 34400 | Erlij D (1976) *Pflügers Archiv*. **363,** 91-93 |
| **Frog skin** | 8700 | Lewis *et al.* (1975) *Physiologist*, **8**, 291 as cited in Powell (1981) *Am. J. Physiol*. **241**, G275-288. |
| **Toad skin** | 763 | Bruus et al. (1976) *Acta. Physiol. Scand.* **97**, 31-47 as cited in Powell Ibid. |
| **Dog distal tubule** | 600 | Boulpaep EL & Seely JF (1971) Ibid. |
| **Rat distal tubule** | 300 | Giebisch G & Malnic G (1968) as cited in: Boulpaep EL & Seely JF Ibid. |
| **Rat distal tubule** | 350 | Malnic G & Giebisch G (1972) *Am. J. Physiol*. **223**, 797-808 |
| **Rabbit colon** | 385 | Anderson & Van Itallie (2009) *CSH Perspectives Biol*, **1**:a002584, 1-16. |
| **Rabbit colon** | 330 | Powell DW (1981) *Am. J. Physiol.* **241,** G275-G288 |
| **Rabbit colon** | 200 | Ghandehari H *et al*. (1997) *J. Pharm. Exp. Thera.* **280**, 747-753 |
| **Freshwater Fish Gill** | 30000 | Tsui *et al*. (2009) *J. Exp. Biol.* **212**, 878-892. |
| **Urinary bladder toad** | 3800 | Reuss & Finn (1974) *J. Gen. Physiol.* **64,** 1-25 |
| **Urinary bladder toad** | 49400 | Erlij D. (1976) Ibid. |
| **Urinary bladder toad** | 83000 | Erlij D. (1976) Ibid. |

**Table S1:** Data sources for meta-analysis of resistance of epithelial cultures

(Adapted from Boulpaep & Seely 1971, Claude & Goodenough 1973 and Powell 1981).
