# Supplementary material for: Freshwater Sponges Have Functional, Sealing Epithelia with High Transepithelial Resistance and Negative Transepithelial Potential
Source: PLoS One. 2010 Nov 29;5(11):e15040. doi: 10.1371/journal.pone.0015040 (PMC2993944; doi:10.1371/journal.pone.0015040)
Supplement: Table S2 — Salt concentrations in culture media. (DOC) [file pone.0015040.s004.doc]

**Table S2**: Salt concentrations in culture media.

| **Medium** | **Concentration (mM)** | **Salt** |
| --- | --- | --- |
| **TER Medium** | 1.5 | MgSO_4_• 7H_2_O |
|  | 1.4 | CaCl_2_• 2H_2_O |
|  | 0.6 | NaHCO_3_ |
|  | 0.45 | KCl |
|  | 0.25 | Na_2_SiO_3_ |
|  | 11-17 | NaCl |
|  | 0.6-0.9 | MgCl_2_• 6H_2_O |
| **Cl - - free TER medium** | 12.7 | Na-gluconate |
|  | 1.95 | MgSO_4_•7H_2_O |
|  | 1.1 | Ca-gluconate |
|  | 0.56 | NaHCO_3_ |
|  | 0.32 | K-gluconate |
|  | 0.25 | Na_2_SiO_3_ |
| **Na^+^ - free TER medium** | 1.5 | MgSO_4_ |
|  | 1.4 | CaCl_2_ |
|  | 0.45 | KHCO_3_ |
|  | 11 | NMDG-Cl |
|  | 0.6 | MgCl_2_ |
| **M-Medium** | 0.5 | MgSO_4_•7H_2_O |
|  | 1.0 | CaCl_2_•2H_2_O |
|  | 0.5 | NaHCO_3_ |
|  | 0.05 | KCl |
|  | 0.25 | Na_2_SiO_3_ |
